# Isolation and Imaging of Microvessels From Brain Tissue

**DOI:** 10.21769/BioProtoc.5410

**Published:** 2025-08-05

**Authors:** Josephine K. Buff, Carolyn R. Bertozzi, Tony Wyss-Coray, Sophia M. Shi

**Affiliations:** 1 Department of Neurology and Neurological Sciences, Stanford University School of Medicine, Stanford, CA, USA; 2 Wu Tsai Neurosciences Institute, Stanford University School of Medicine, Stanford, CA, USA; 3 Department of Chemistry, Stanford University, Stanford, CA, USA; 4 Stanford Chemistry, Engineering and Medicine for Human Health (ChEM-H), Stanford University, Stanford, CA, USA; 5 Howard Hughes Medical Institute, Stanford University, Stanford, CA, USA; 6 The Phil and Penny Knight Initiative for Brain Resilience, Stanford University, Stanford, CA, USA

**Keywords:** Vasculature, Brain endothelial cells, Blood–brain barrier, Microvessel isolation, Neurodegeneration, Aging

## Abstract

Proper brain function depends on the integrity of the blood–brain barrier (BBB), which is formed by a specialized network of microvessels in the brain. Reliable isolation of these microvessels is crucial for studying BBB composition and function in both health and disease. Here, we describe a protocol for the mechanical dissociation and density-based separation of microvessels from fresh or frozen human and murine brain tissue. The isolated microvessels retain their molecular integrity and are compatible with downstream applications, including fluorescence imaging and biochemical analyses. This method enables direct comparisons across species and disease states using the same workflow, facilitating translational research on BBB biology.

Key features

• The protocol employs mechanical dissociation and density-based separation to isolate microvessels from brain tissues.

• The protocol was used to study molecular changes in brain microvessels in neurodegeneration and aging.

• Validated downstream applications of this method include fluorescence imaging, RNA sequencing, proteomics, western blotting, and ELISA.

• The protocol can be applied to fresh and frozen human and murine brain samples.

## Graphical overview


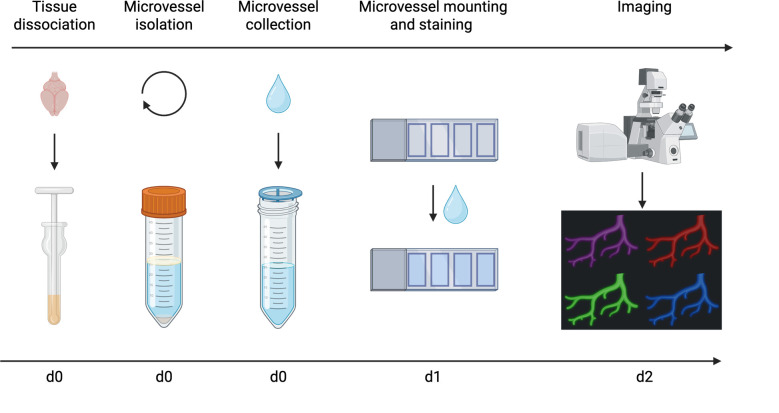


**Overview of microvessel isolation and imaging procedure**

## Background

The blood–brain barrier (BBB) is a highly selective neurovascular interface that tightly regulates the exchange of substances between the blood and brain parenchyma [1]. Its primary physiological function involves maintaining central nervous system (CNS) homeostasis by supplying the brain with necessary nutrients while protecting neural cells from bloodborne pathogens and toxic soluble factors [1–3]. The BBB is formed by different cell types and structural components, including endothelial cells, astrocytic end-feet, pericytes, the basement membrane, and a luminal glycocalyx layer [3,4]. Brain endothelial cells (BECs), which form the inner lining of the cerebrovasculature, are structurally and functionally distinct from endothelial cells in peripheral organs [5]. BECs form continuous, non-fenestrated capillaries with tight and adherens junctions that severely restrict paracellular diffusion across the BBB [1,6]. The permeability of the BBB is further reduced by limited fluid-phase transcytosis and active efflux transporters that eject unwanted substances from BECs [6]. The luminal side of BECs is lined by the glycocalyx, a negatively charged layer formed by glycans and glycoconjugates, which further supports BBB properties [4,7,8]. BBB integrity is an important factor for brain health, and its disruption is implicated in the pathogenesis of several neurodegenerative diseases, including Alzheimer’s disease and multiple sclerosis [2]. BBB dysfunction involves a range of pathological alterations, including increased inflammation, pericyte loss, oxidative stress, and alterations in protein and glycocalyx composition [2,4].

Detailed molecular and cellular characterization of brain microvessels is crucial for our understanding of BBB physiology and dysfunction in disease states. Here, we present a robust and reproducible protocol for microvessel isolation that utilizes mechanical dissociation and density gradient-based separation of parenchymal and vascular cells for subsequent microvessel analyses. Our protocol builds on previous methods [9,10] and enables a multitude of downstream analyses, including RNA sequencing (RNA-seq), proteomics, fluorescence imaging of cell surface and intracellular markers, western blotting, and ELISA. In Shi et al. (2025) [4], we utilized this protocol to interrogate molecular changes in the brain vasculature in aging and neurodegenerative disease, including alterations in cell surface glycosylation, reactive oxygen species (ROS), and protein abundance. Unlike approaches that isolate individual endothelial cells using transgenic markers (e.g., Tie2-eGFP) [11] or antibody-based selection (e.g., CD31^+^ sorting) [12], our protocol enables the study of intact microvascular units, allowing for the investigation of intercellular interactions and composite BBB properties. In addition, this method avoids harsh enzymatic dissociation or overly aggressive mechanical methods to isolate microvessels [13,14], which can result in molecular changes to the analyzed tissue [9]. This protocol has been successfully applied to fresh and frozen tissue of both murine and human origin.

## Materials and reagents

**Biological materials**

1. C57BL/6J mice (Jackson Laboratory, catalog number: 000664)

**Reagents**

1. Bovine serum albumin (BSA) (Thermo Fisher Scientific, catalog number: 50-253-900)

2. Phosphate buffered saline (PBS) (Thermo Fisher Scientific, Gibco^TM^, catalog number: 10010049)

3. cOmplete^TM^, EDTA-free protease inhibitor (PI) cocktail (Thermo Fisher Scientific, Roche, catalog number: 11873580001)

4. HBSS, calcium, magnesium, no phenol red (Thermo Fisher Scientific, Gibco^TM^, catalog number: 14025092)

5. Dextran from *Leuconostoc mesenteroides*, average molecular weight 60,000–75,000 g/mol (Millipore Sigma, Sigma-Aldrich, catalog number: D8821-100G)

6. 32% paraformaldehyde (formaldehyde) aqueous solution (PFA) (Thermo Fisher Scientific, Electron Microscopy Sciences, catalog number: 50-980-495)

7. Poly-D-Lysine (PDL) (Thermo Fisher Scientific, Gibco^TM^, catalog number: A3890401)

8. TRIS-buffered saline (TBS), 10×, pH 7.4 (Thermo Fisher Scientific, Thermo Scientific Chemical, catalog number: J60764.K2)

9. TWEEN^®^ 20 (Millipore Sigma, Sigma-Aldrich, catalog number: P1379-100ML)

10. Normal donkey serum (Jackson ImmunoResearch, catalog number: 017-000-121)

11. Triton^TM^ X-100 solution (TX-100) (Millipore Sigma, catalog number: 93443)

12. ProLong^TM^ gold antifade mountant (Thermo Fisher Scientific, Invitrogen^TM^, catalog number: P36934); alternatively, VECTASHIELD HardSet^TM^ antifade mounting medium with DAPI, 10 mL (Thermo Fisher Scientific, Vector Laboratories, catalog number: NC9029229)

**Solutions**

1. 1% BSA in PBS (see Recipes)

2. 1% BSA + 1× protease inhibitor (PI) in PBS (see Recipes)

3. 32% Dextran solution (see Recipes)

4. TBS-T (see Recipes)

5. 4% PFA solution (see Recipes)

6. Blocking solution (see Recipes)

**Recipes**

**1. 1% BSA in PBS**

Prepare in advance. Add 5 g of BSA to a sterile 500 mL bottle with 1× PBS and shake to mix. BSA will dissolve into PBS overnight. Store at 2–8 °C for up to four weeks.

**Table:** 

Reagent	Final concentration	Quantity or Volume
BSA	1% (w/v)	5 g
PBS	1×	500 mL
Total	n/a	500 mL

**2. 1% BSA + 1**× **protease inhibitor (PI) in PBS**

Prepare and aliquot 25× PI according to the manufacturer’s instructions. Thaw a vial of 25× PI and add 200 μL of 25× PI to 4,800 μL of 1% BSA in PBS. Prepare 5 mL per sample. Store on ice and make fresh before every microvessel isolation.

**Table:** 

Reagent	Final concentration	Quantity or Volume
25× protease inhibitor	1×	200 μL
1% BSA in PBS (from Recipe 1)	n/a	4,800 μL
Total	n/a	5,000 μL

**3. 32% Dextran solution**

Prepare in advance. Add HBSS to 100 g of Dextran from *Leuconostoc mesenteroides* for a final volume of 312.5 mL. Add a clean magnetic stirring rod and stir at room temperature until dissolved. Can be stored at 4 °C for up to 3 months.

**Table:** 

Reagent	Final concentration	Quantity or Volume
Dextran	32% (w/v)	100 g
HBSS	n/a	Fill to 312.5 mL
Total	n/a	312.5 mL

**4. TBS-T**

Dilute 100 mL of 10× TBS in 899.5 mL of ultrapure water and add 500 μL of Tween 20. Invert until completely mixed. Store for up to one year at room temperature.

**Table:** 

Reagent	Final concentration	Quantity or Volume
Tween 20	0.05%	0.5 mL
10× TBS	1×	100 mL
Ultrapure H_2_O	n/a	899.5 mL
Total	n/a	1,000 mL

**5. 4% PFA solution**

Dilute 3.75 mL of 32% PFA in 26.25 mL of 1× PBS at room temperature. Make fresh before use.

**Table:** 

Reagent	Final concentration	Quantity or Volume
32% PFA	4%	3.75 mL
PBS	n/a	26.25 mL
Total	n/a	30 mL

**6. Blocking solution**

Add 3% normal donkey serum (NDS) and 0.3% TX-100 to TBS-T. Make enough for blocking and primary and secondary antibody staining steps. Calculate approximately 200 μL total per sample (volume will vary depending on the size of the hydrophobic rectangle). Make fresh before use.

*Note: TBS-T can be replaced with TBS or PBS if sample permeabilization is not needed.*

**Table:** 

Reagent	Final concentration	Quantity or Volume
NDS	3%	45 μL
TX-100	0.3%	4.5 μL
TBS-T (Recipe 4)	n/a	1,450.5 μL
Total	n/a	1,500 μL

**Laboratory supplies**

1. Corning^®^ 15 mL centrifuge tubes (Millipore Sigma, catalog number: CLS430790-500EA)

2. Corning^®^ 50 mL centrifuge tubes (Millipore Sigma, catalog number: CLS4558-300EA)

3. VWR^®^ disposable Petri dishes, 60 × 15, mono Petri dishes, fully stackable (EO sterilization) (VWR International, catalog number: 25384-168)

4. Corning^®^ tissue culture–treated culture dishes, 150 mm × 25 mm (Corning, Millipore Sigma, catalog number: CLS430599-60EA)

5. VWR^®^ razor blades (VWR International, catalog number: 55411-050)

6. Corning^®^ cell strainer, pore size 40 μm (Corning, Millipore Sigma, catalog number: CLS431750-50EA)

7. Kimberly-Clark Professional^TM^ Kimtech Science^TM^ Kimwipes^TM^ delicate task wipers, 1 ply (Kimberly-Clark Professional, Thermo Fisher Scientific, catalog number: 06-666)

8. Snap-cap low-retention microcentrifuge tubes (Thermo Fisher Scientific, catalog number: 3448PK)

9. Fisherbrand^TM^ Superfrost^TM^ Plus microscope slides (Thermo Fisher Scientific, catalog number: 12-550-15)

10. ImmEdge^®^ hydrophobic barrier PAP pen (Vector Laboratories, catalog number: H-4000)

11. Fisherbrand^TM^ Premium cover glasses (Thermo Fisher Scientific, catalog number: 12-548-5A)

12. Fisherbrand^TM^ aluminum foil, standard-gauge roll (Thermo Fisher Scientific, catalog number: 01-213-102)

13. TipOne^®^ filter tips, 1,000 μL graduated (USA Scientific, catalog number: 1125-7810)

14. TipOne^®^ filter tips, 200 μL graduated (USA Scientific, catalog number: 1120-8810)

15. Nunc^TM^ serological pipettes, 5 mL (Thermo Fisher Scientific, catalog number: 170355N)

16. Nunc^TM^ serological pipettes, 10 mL (Thermo Fisher Scientific, catalog number: 170356N)

17. Nunc^TM^ serological pipettes, 25 mL (Thermo Fisher Scientific, catalog number: 170357N)

## Equipment

1. Wheaton^®^ 357424 glass 7 mL Tenbroeck tissue grinder set, grinding chamber O.D. × L: 16 × 82 mm (case of 2) (Dounce homogenizer) [DWK Life Sciences Inc. (Wheaton), catalog number: 357424]

2. CAPP motorized pipette controller, 0.1–100 mL (Pipette.com, catalog number: PA-100)

3. Centrifuge 5810/5810 R (Eppendorf, catalog number: 022625004)

4. Corning^TM^ LSE^TM^ low-speed orbital shaker (Corning, Thermo Fisher Scientific, catalog number: 10-320-813)

5. Nalgene^TM^ polycarbonate Erlenmeyer flasks (Thermo Fisher Scientific, catalog number: 4103-0250)

6. Confocal laser-scanning microscope (Zeiss, catalog number: LSM880)

## Software and datasets

1. ZEN Microscopy Software (Carl Zeiss Microscopy GmbH, Version 3.079.0006)

2. ImageJ (National Institutes of Health and the Laboratory for Optical and Computational Instrumentation, Version 1.54p or higher)

3. GraphPad Prism (Graphpad Software, Inc., Version 10.4.1 or higher)

## Procedure

**A. Before you begin**

1. Prepare the following items the day before starting the microvessel preparations.

a. Prepare PDL-coated slides by pipetting 300 μL of PDL onto each slide, making sure to cover most of the slide surface. Cover slides and incubate at room temperature overnight. The next day, wash off excess PDL with ddH_2_O and allow slides to dry at room temperature.

b. Prepare 1% BSA in PBS and 32% Dextran solution the day before (see Recipes).

c. Place a 500 mL bottle of PBS into a 4 °C refrigerator to cool down overnight.

2. Prepare the following items before beginning the microvessel preparations:

a. Label one microcentrifuge tube, one 15 mL conical tube, one 50 mL conical tube, and one 40 μm strainer per sample and set aside.

b. Prepare 1% BSA + 1× PI in PBS (see Recipes) and store on ice.

c. Fill the previously labeled 15 mL conical tubes with 10 mL of 32% Dextran solution and place on ice.

d. Place Dounce homogenizers on ice.

e. Cut 5 mm off the ends of 1,000 μL pipette tips, preparing one modified tip per sample.

**B. Tissue dissociation**

1. For tissue preparation, see General notes.

2. Place brain tissue in 0.5 mL of 1% BSA + 1× PI in PBS in a 60 mm Petri dish on ice.

*Note: The volumes of 1% BSA + 1× PI in PBS are specified for a single mouse hemisphere (approximately 0.2 mg). Adjust volumes accordingly based on tissue size.*

**Figure 1.**
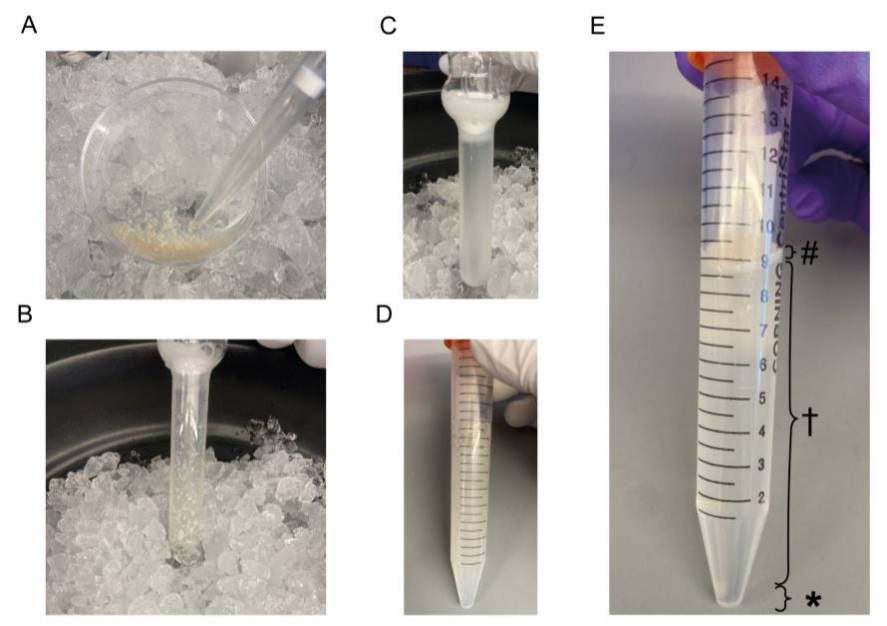
Tissue dissociation and microvessel isolation. (A) Chopped brain tissue with a cut 1000 µL pipette tip. (B) Dounce homogenizer with chopped brain tissue before dissociation. (C) Dounce homogenizer after dissociation. (D) Dissociated tissue mixed with 32% Dextran solution before centrifugation. (E) Dissociated tissue mixed with 32% Dextran solution after centrifugation separated into different layers: (#) myelin layer, () parenchymal layer, and (*) vascular layer.

3. Using a razor blade, chop brain tissue into ~1 mm^3^ pieces. The pieces should be small enough to fit through precut 1,000 μL pipette tips but large enough not to fit through an uncut 1,000 μL pipette tip (Figure 1A).

4. Using the precut 1,000 µL pipette tip, transfer the chopped brain tissue to the Dounce homogenizer tube on ice.

*Note: It is helpful to use two 101–1,000 μL pipettes in this section: one for transferring tissue with the precut 1,000 µL pipette tip to maximize sample transfer, and another for transferring 1% BSA + 1× PI in PBS for washing.*

5. Wash the razor blade and Petri dish with an additional 1 mL of 1% BSA + 1× PI in PBS and transfer as much tissue as possible to the Dounce homogenizer tube using the same precut 1,000 μL pipette tip.

6. Gently place the Dounce homogenizer pestle in the tube and slowly push it toward the bottom. To avoid compressing all the tissue at the bottom, carefully move the pestle up and down to disperse the tissue pieces along the sides of the tube before pushing the pestle all the way to the bottom (Figure 1B).

7. To homogenize the tissue, gently move the pestle up and down along the complete length of the Dounce homogenizer approximately 20 times. Avoid creating bubbles by pulling the pestle out of the dounce completely. After ~20 strokes, the tissue pieces should be completely dissociated, with only the microvessels remaining intact (Figure 1C).

**Critical:** Avoid twisting the pestle, as this fractures the fragile microvessels.

**Critical:** Keep the Dounce homogenizer submerged in ice as much as possible during homogenization or conduct homogenization in a cold room to help preserve the molecular integrity of microvessels.

8. Remove the pestle and put it to the side. Using a 5 mL serological pipette, transfer the contents of the Dounce homogenizer to the 15 mL conical tube filled with 10 mL of 32% Dextran solution.

9. With a 1,000 μL pipette, wash the pestle and dounce homogenizer with 1 mL of 1% BSA + 1× PI in PBS and transfer to the same 15 mL conical tube filled with 32% Dextran solution.

*Note: Wash the Dounce homogenizer with PBS between every sample and avoid using the same Dounce homogenizer for different conditions without extensive washing in between (see General notes 1).*

**C. Microvessel isolation**

1. Mix the dissociated tissue with the 32% Dextran solution by inverting the 15 mL conical tube roughly 20 times (Figure 1D).

**Critical:** Make sure that the contents are fully homogenized before moving on.

2. To separate the vascular fraction, centrifuge the 15 mL conical tubes at 4,400× *g* for 25 min at 4 °C with slow deceleration.

a. After centrifugation, the contents will have separated into three distinct layers: a myelin layer (top), the parenchymal cells within the dextran (middle), and the vascular fraction (bottom) (Figure 1E).

3. To isolate the microvessel with minimal contamination, the layers need to be separated carefully. First, remove the myelin layer using a 5 mL serological pipette. Any remaining myelin can be removed using a Kimwipe to clean the insides of the conical tube.

4. Remove the parenchymal fraction with a 10 mL serological pipette, leaving approximately 1 mL of supernatant in the conical tube.

**Critical:** Make sure the vascular pellet is not disturbed to avoid losing microvessels in the isolation process.

**D. Microvessel collection**

**Figure 2.**
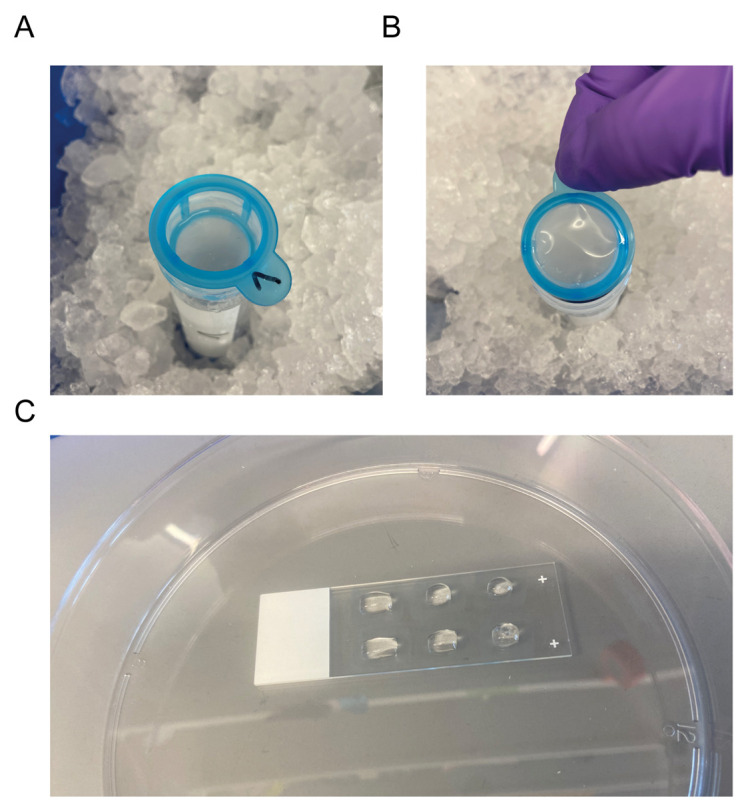
Microvessel collection and staining. (A) Strainer on top of a 50 mL conical tube. (B) Position of the strainer for the microvessel collection. (C) Microvessels plated on PDL-coated slides in hydrophobic squares.

1. Before collecting the microvessels, place labeled 40 μm strainers on 50 mL conical tubes. Wet the 40 μm strainer using 5 mL of 1% BSA in PBS, making sure to wet all mesh areas of the strainer.

2. Resuspend the vascular pellet in the 15 mL conical tube with 1 mL of 1% BSA + 1× PI in PBS and add to the strainer (Figure 2A).

3. Wash the microvessels with 30 mL of cold PBS using a 25 mL serological pipette.

*Note: Add the cold PBS slowly, being careful not to overflow the 40 μm strainer or dissociate the microvessels.*

4. To fix the microvessels, place the 40 μm strainer in a 150 mm Petri dish filled with 4% PFA solution. Cover the Petri dish with aluminum foil and place it on a gentle laboratory shaker for 15 min at room temperature.

**Critical:** Make sure no air is trapped beneath the 40 μm strainers, as this impacts the microvessel fixation.

*Note: For downstream applications that do not require fixation (e.g., live imaging, RNA-seq, proteomics, or western blot), omit steps D4–5. See General notes for more information.*

5. After 15 min, move the 40 μm strainers onto the same empty 50 mL conical tubes and wash with 20 mL of cold PBS.

6. To collect the microvessels, move 40 μm strainers to the previously labeled 50 mL conical tubes. Invert the 40 µm strainers and collect the microvessels by carefully washing the inverted 40 μm strainers with 20 mL of 1% BSA in PBS (Figure 2B).

*Note: Make sure to wash all sides of the strainer and add the 1% BSA in PBS slowly so the liquid goes through the strainer mesh and not over the plastic sides. Be careful to collect all liquid into the conical tube. Inspect the strainer to make sure the majority of microvessels have been collected.*

7. Centrifuge the 50 mL conical tube with the collected microvessels at 2,000× *g* for 15 min at 4 °C with slow deceleration.

8. Take off as much supernatant as possible without disturbing the microvessel pellet. To transfer the microvessels to labeled microcentrifuge tubes, use the remaining supernatant (approximately 100–400 μL) to resuspend the pellet and then transfer the microvessel containing supernatant to the microcentrifuge tube using a 1,000 μL pipette.

**Pause point**: The microvessels can be stored at 4 °C for up to 2 weeks.

*Note: The resulting microvessels can now be used for different downstream applications, including RNA sequencing, western blot, proteomics, and ELISA. For representative examples, see [4] and [15]. See General notes for more information.*

**E. Microvessel mounting and staining**

1. Prepare the previously coated PDL slides by drawing up to six rectangles per slide with the hydrophobic pen. Place the PDL slides into a 150 mm Petri dish and wait for the hydrophobic barriers to dry before proceeding.

2. Gently resuspend the microvessels in the microcentrifuge tubes by gently pipetting up and down with a 1,000 µL pipette.

3. Add approximately 30 μL of resuspended microvessels per rectangle (Figure 2C).

*Note: The amount of liquid will vary depending on the rectangle size.*

4. Loosely cover the 150 mm Petri dish with aluminum foil and let the slides dry overnight at room temperature.

5. The next day, add 100 µL of blocking solution to the rectangles, loosely cover with aluminum foil, and incubate for 1 h at room temperature.

*Note: The amount of liquid will vary depending on the rectangle size.*

**Critical:** When adding or removing liquid from the slides, carefully place the pipette tip at the same corner each time and pipette very slowly. The slides can be carefully tilted to simplify liquid transfer, but take care to avoid liquid spillover, as this could lead to microvessel loss.

**Figure 3.**
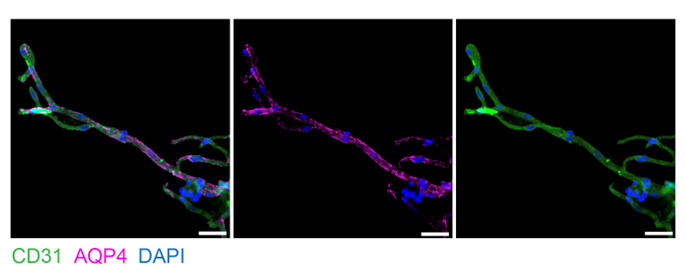
Imaging of microvessels isolated from fresh murine brain tissue. Microvessels imaged at 40× magnification, stained with AQP4 for astrocytic end-feet, CD31 for endothelial cells, and DAPI for nuclei. Scale bar = 25 μm.

6. Carefully remove blocking solution and add primary antibodies diluted in 30 μL of blocking solution per rectangle. Loosely cover with aluminum foil and incubate for 1 h at room temperature.

a. In this experiment, the following antibodies were used at a 1:250 dilution: AQP4 (Millipore, catalog number: AB2218) and CD31 (R&D Systems, catalog number: AF3628) (Figure 3).

7. Remove primary antibodies and wash three times with 30 μL of TBS-T for 5 min each.

*Note: TBS-T can be replaced with TBS or PBS in all washing steps if sample permeabilization is not needed.*

8. Add secondary Alexa Fluor-conjugated antibodies diluted in 30 µL of blocking solution per rectangle. Loosely cover with aluminum foil and incubate for 1 h at room temperature.

**Critical:** Protect slides from light from this step onward to prevent loss of fluorescence signal.

a. In this experiment, the following antibodies were used at a 1:500 dilution: donkey anti-goat IgG (H+L) highly cross-adsorbed secondary antibody, Alexa Fluor^TM^ Plus 488 (Invitrogen, catalog number: A32814) and donkey anti-rabbit IgG (H + L) highly cross adsorbed secondary antibody, Alexa Fluor^TM^ 555 (Invitrogen, catalog number: A31572).

9. Remove secondary antibodies and wash two times with 30 μL of TBS-T and one time with PBS for 5 min each.

10. Remove the final PBS wash and let microvessels dry.

11. Mount by adding 20 μL of mounting media to each rectangle and adding a coverslip.

*Note: Avoid air bubbles when mounting.*

12. Dry the slides at room temperature overnight in a light-protected ventilated area (i.e., drawer).

13. Store slides at 4 °C protected from light.

**F. Imaging**

1. Image using a confocal laser-scanning microscope (Zeiss LSM880) equipped with the ZEN software, 40–63× objectives (oil immersion), and lasers with the 647, 555, 488, and 405 nm excitation wavelengths.

*Note: Alternatively, use a comparable confocal microscope with affiliated software.*

**Figure 4.**
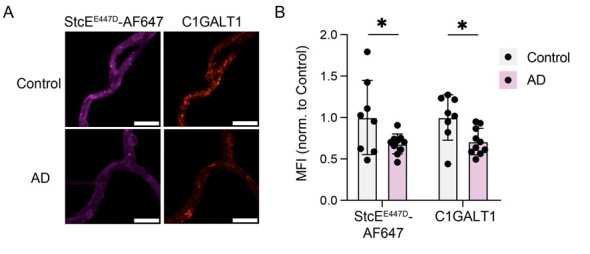
Imaging of microvessels isolated from frozen human brain tissue. (A) Microvessels imaged at 40× magnification, stained with StcE^E447D^-AF647 for mucins and C1GALT1. Scale bar = 10 μm. (B) Quantification of (A) (n = 8 control and 10 AD samples, two-sided t-test; mean ± s.e.m.) (adapted from Shi et al., Nature (2025) [4], Figure 2k–l).

2. Adjust the settings as desired and keep consistent for all conditions for quantitative comparisons.

3. Capture microvessels by centering a Z-stack around the region of interest with optimal slice thickness.

4. For each sample, capture several fields of view to encompass enough single-plane microvessels for quantification (Figures 3 and 4).

## Data analysis

1. Using the ImageJ software, perform Z-projections of the acquired images by going to *Image* → *Stacks* → *Z-Project* and selecting *Max Intensity.*

2. To select a region of interest (ROI) (e.g., single-plane microvessels), duplicate the image using *Image* → *Duplicate* and select a single channel that is consistent across conditions.

3. With this duplicated image, use *Image* → *Adjust* → *Threshold* and change the maximum and minimum threshold manually or with an automated thresholding algorithm until only the microvessels are highlighted. Limit ROI to single-plane microvessels for quantification if desired.

4. Then, select the highlighted microvessels by using *Analyze* → *Analyze Particles*.

5. Apply the selected ROI to the Z-projected image by selecting *More* → *OR (combine)* in the ROI manager window.

6. Measure the intensity of the signal within the ROI for each channel by using *Analyze* → *Measure*.

*Note: Refer to Shi et al. (2025) for the statistical analysis.*

## Validation of protocol

This protocol (or parts of it) has been used and validated in the following research article:

- Shi et al. [4]. Glycocalyx dysregulation promotes blood-brain barrier dysfunction in ageing and disease. *Nature* (Figure 1G–O; Figure 2F–L; Figure 3B–D, M–N; Figure 4B–F; Extended Data Figure 2A–J; Extended Data Figure 4A–F; Extended Data Figure 7D–E; and Extended Data Figure 9A–D).

## General notes and troubleshooting

**General notes**

1. Plan each microvessel isolation with appropriate control and experimental conditions, alternating between conditions to avoid potential biases if blinding is not feasible.

2. When using fresh brain samples, perfuse anesthetized mice with 30 mL of cold PBS. Remove the brain, divide into hemispheres, and place in cold 1% BSA + 1× PI in PBS in 24-well plates on ice.

3. To use frozen brain samples for microvessel isolation, let the sample thaw just enough for chopping on ice before proceeding with the protocol.

*Note: The yield of isolated microvessels may decrease when using frozen brain samples.*

4. If processing samples from different conditions, avoid cross-contamination, i.e., use different Dounce homogenizers for each condition or wash extensively between samples.

5. Until microvessel fixation, perform all steps on ice or at 4 °C to avoid degradation or molecular alterations.

6. For downstream applications that do not require fixation (e.g., live imaging, RNA-seq, proteomics, or western blot), include the following adjustments to the protocol:

a. For downstream RNA-seq and proteomic analyses, include RNase or protease inhibitors in buffers as required.

b. Skip fixation steps D4–5 and proceed directly to the collection of the microvessels.

c. Following step D8, additional sample handling procedures for microvessel live imaging, proteomics, or western blot are described in Shi et al. [4] and Shi et al. [15].

**Troubleshooting**

Problem 1: No microvessel pellet after step C2.

Possible cause: Brain tissue may have been over-homogenized.

Solution: Make sure the pestle is loosely fit. Make sure chopping does not result in too fine tissue chunks. Try douncing with fewer strokes until the tissue is just homogenized and avoid excessively twisting the pestle.

Problem 2: Low yield of microvessels after step D8.

Possible cause: Microvessels might have been lost in the collection process.

Solution: Be sure to wash all mesh parts of the strainer, including the sides, to collect all microvessels. Additionally, be careful to wash through the strainer mesh without the liquid running off the outside of the strainer.

Problem 3: Low yield of microvessels during imaging.

Possible cause: Microvessels are lost during staining or washing steps.

Solution: Always use one corner of the hydrophobic squares to slowly add and remove liquid to avoid disturbing microvessels as much as possible. Check for the presence of microvessels before and after staining/washing steps.
